# Paratesticular Serous Papillary Carcinoma of High Grade and Malignant Potential: A Rare Case with a Role for Adjuvant Therapy

**DOI:** 10.7759/cureus.2188

**Published:** 2018-02-13

**Authors:** Jacob Lifton, Saum Ghodoussipour, Guang-Qian Xiao, Tanya Dorff, Jeffrey Loh-Doyle, Stuart D Boyd

**Affiliations:** 1 Medical Student, Lac+usc Medical Center and Chla, Keck School of Medicine at Usc; 2 Department of Urology, Keck School Of Medicine at USC; 3 Department of Pathology, Keck School Of Medicine at USC; 4 Division of Oncology, Keck School Of Medicine at USC

**Keywords:** testicular cancer, serous papillary carcinoma, adjuvant chemotherapy, müllerian testicular tumor

## Abstract

Paratesticular serous papillary carcinomas are very rare, with less than 40 cases reported in the literature. These neoplasms are Müllerian in origin, and more commonly seen as epithelial-type ovarian cancer. Given the rarity of this tumor in men, staging and recommended treatment options do not exist. Herein, we present the case of a 35-year-old male with high-grade invasive serous papillary carcinoma. He was diagnosed after left radical orchiectomy for paratesticular mass and subsequently treated with adjuvant chemotherapy according to existing recommendations for its ovarian counterpart. Chemotherapy was well tolerated and surveillance imaging has shown no evidence of disease. This case suggests a potential role for adjuvant therapy in patients with high-grade paratesticular serous papillary carcinoma.

## Introduction

Testicular cancer represents one of the most common malignancies in young men in the United States. The vast majority are germ cell tumors, typically classified as either seminomatous or non-seminomatous tumors [1]. Several unusual non-germ cell subtypes have been reported in the literature, including serous papillary carcinoma [2]. The majority of these cases have been treated primarily with orchiectomy, with final pathology showing either low-grade or borderline histology [3]. A thorough literature review was conducted and revealed only two reports of tumors demonstrating high-grade histologic features, both of which were metastatic at presentation [4,5]. Given the rarity of serous papillary carcinoma in men—particularly that of high-grade histology—staging and standardized treatment options do not yet exist. Herein, we present the case of a 35-year-old male with invasive, nonmetastatic high-grade serous papillary carcinoma treated with radical orchiectomy and adjuvant chemotherapy.

## Case presentation

**Figure 1:**
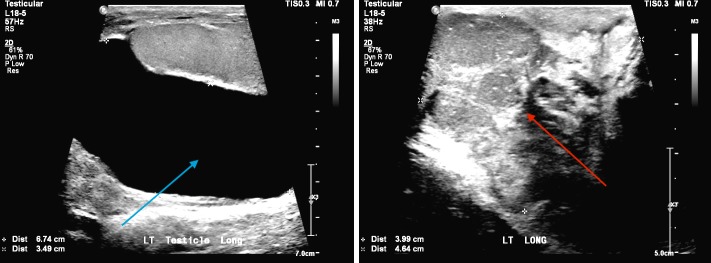
Ultrasound of the left testis showing hydrocele (left, blue arrow), and heterogeneous paratesticular mass (right, red arrow).

The patient was a previously healthy 35-year-old male who had been followed for several years for an asymptomatic left hydrocele. He noted an increase in the size of the hydrocele and nodularity to his scrotum over the course of a year, and was subsequently referred to Urology by his primary physician for further evaluation. On initial assessment, the left testicle revealed a hydrocele, along with a very firm and nodular mass superiorly in the scrotum. His physical exam was otherwise benign, with no gynecomastia and a normal right testicle. An ultrasound of the left testicle was performed, showing a lobulated, heterogeneous left paratesticular mass measuring 4.1 x 4.7 cm with heterogeneous blood flow (Figure 1).

**Figure 2:**
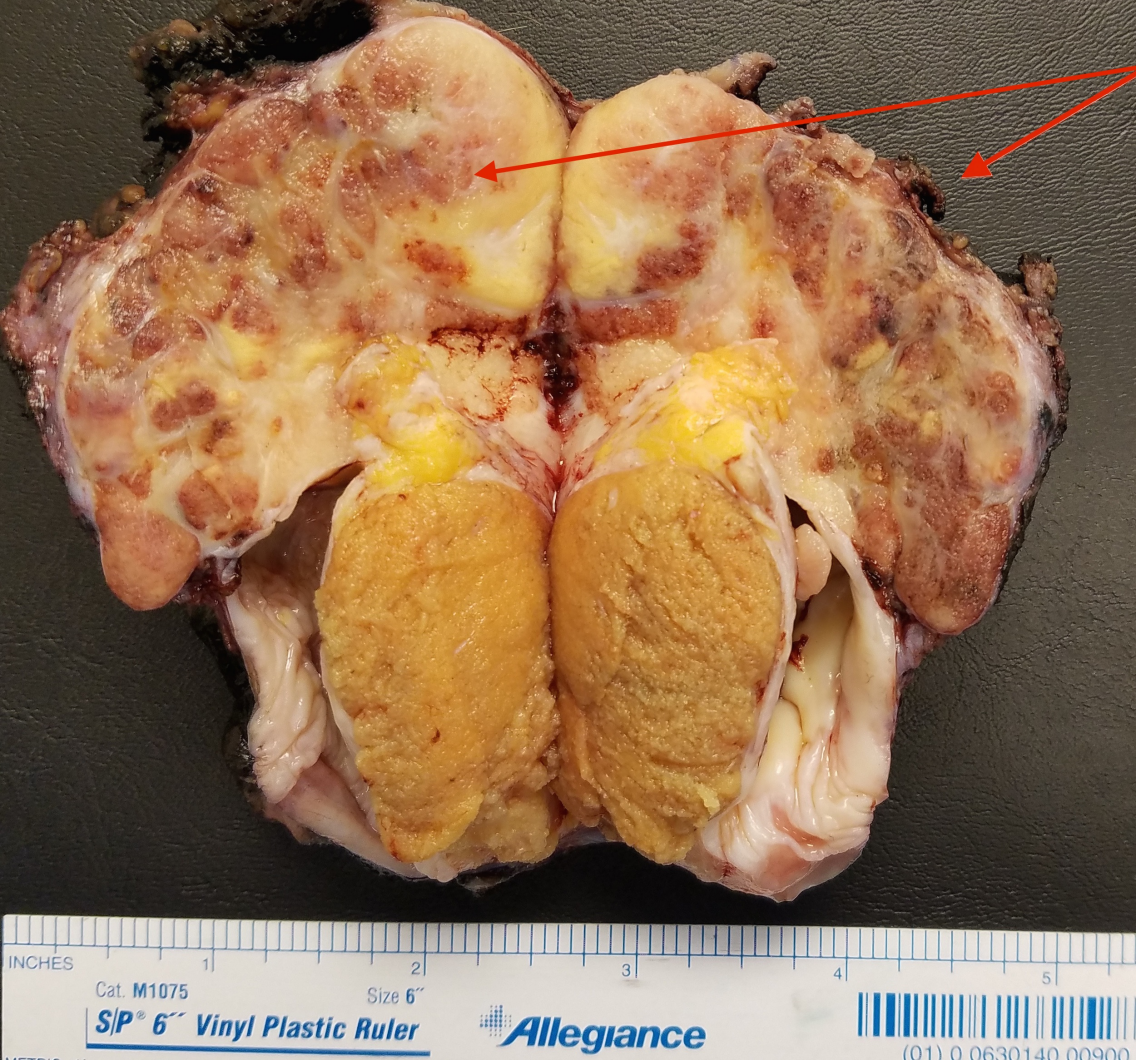
Bisected gross specimen showing nodular tumor originating in testiculo-epididymal groove (red arrows indicate the bisected halves of the tumor).

Computed tomography (CT) scan of the abdomen and pelvis was negative for metastatic disease. The patient was subsequently taken to the operating room for left radical orchiectomy. Intraoperative findings revealed a left hydrocele with a superiorly-positioned mass that was adherent to the spermatic cord, left testicle and scrotal skin. The mass, testicle and overlying scrotal skin were resected en-bloc, and a prosthesis was placed in the left hemi-scrotum. Final pathology of the gross specimen revealed a 6.6 cm tumor emanating from the testiculo-epididymal groove and invading into the base of the spermatic cord and scrotal wall (Figure 2).

**Figure 3:**
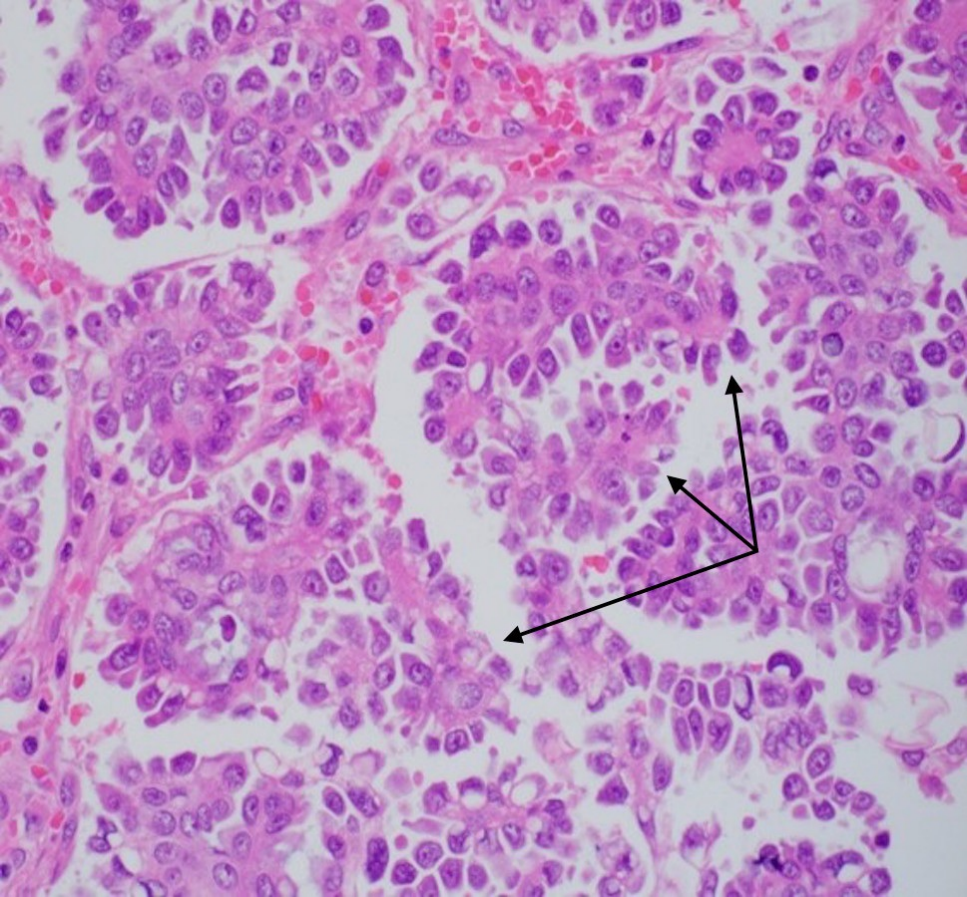
Specimen histology showing papillary structures (black arrows) surrounding fibrovascular cores, lined by high grade tumor cells.

The epididymis, testicular parenchyma, and hydrocele sac were uninvolved. Specimen histology showed high-grade tumor cells forming papillary structures that surrounded fibrovascular cores (Figure 3).

**Figure 4:**
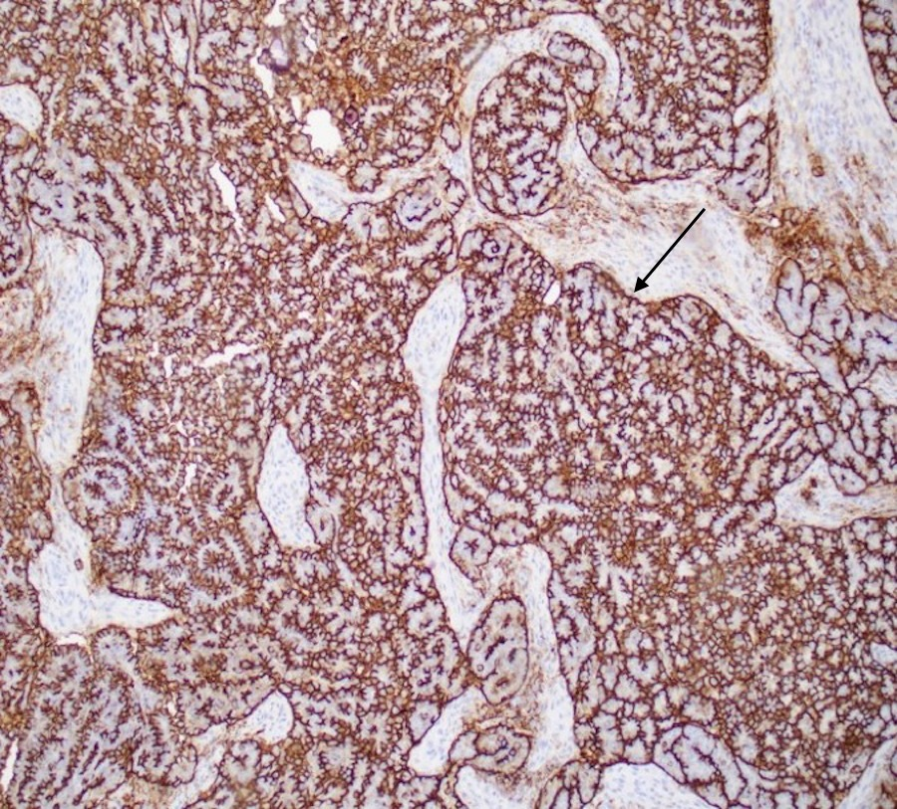
Immunohistochemical staining positive for CA-125 (stain indicated by arrow). CA-125: Cancer Antigen 125

**Figure 5:**
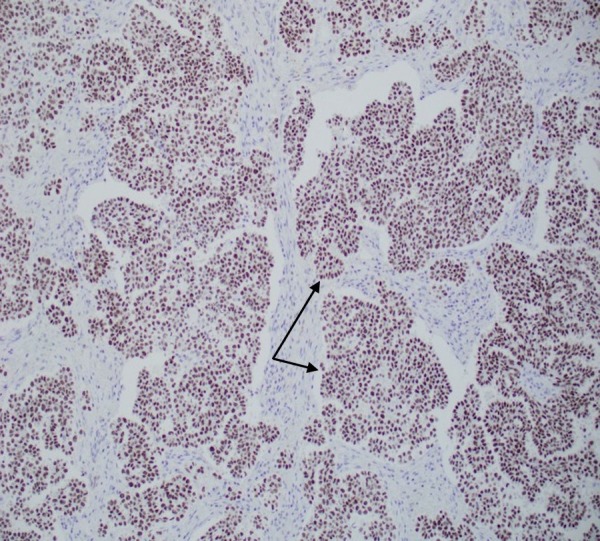
Immunohistochemical staining positive for WT-1 (stain indicated by arrows). WT-1: Wilms Tumor Protein

Lymphovascular invasion was present on histological examination, and resection margins were deemed negative in the final pathology report. Immunohistochemical staining was positive for Paired Box Gene 8 (PAX8), Wilms Tumor Protein (WT1), and Cancer Antigen 125 (CA-125), supporting the diagnosis of high-grade paratesticular serous papillary carcinoma (Figures 4, 5).

The patient recovered uneventfully from his surgery, but given the high-grade pathology of his disease, he was referred to Medical Oncology for further assessment and adjuvant treatment. While there is little literature about the treatment of testicular serous papillary carcinoma, high-quality evidence exists for serous papillary cancer in the ovary—the most similar malignant process to that of our patient. Evidence from the literature demonstrates that in cases of ovarian serous papillary carcinoma, adjuvant chemotherapy improves survival and reduces the risk of recurrence compared with no adjuvant chemotherapy [6]. As such, the patient was offered and started on carboplatin and paclitaxel, based on the most recent Gynecological Cancer InterGroup (GCIG) consensus. The patient suffered an infusion reaction during his first cycle of chemotherapy, but this resolved after increasing his dose of concomitant dexamethasone. He completed a total of four cycles of this chemotherapy regimen over the course of two months. No other adverse reactions were documented.

After his final round of chemotherapy, the patient was deemed to be disease-free, supported by a negative CT scan of the chest, abdomen, and pelvis. As of his most recent follow-up appointment three months after his last treatment, the patient continues to do well with no evidence of disease on imaging and a serum CA-125 level of 14.1 U/mL (Reference range 0-35 U/L).

## Discussion

Müllerian-derived serous papillary carcinoma is a rare malignancy in males and its origin is not fully understood. Certain remnants of the Müllerian ducts do exist in the male reproductive tract, including the appendix testis, the appendix epididymis, the vas aberrans, and the paradidymis. The appendix testis is found along the testiculo-epididymal groove in most men [7]. As such, the location of our patient’s tumor at the superior pole of the testis—immediately adjacent to the testiculo-epididymal groove—may be consistent with a tumor originating from the appendix testis.

Whereas ovarian cancer represents the leading cause of gynecologic death in women [8], nearly all of the reported cases of serous papillary carcinoma in men have been of borderline or low-grade histology, with a rare potential for metastasis [2,4,5]. Interestingly, our patient’s tumor was found to have high-grade features and involved the spermatic cord and scrotal wall, but showed no signs of metastasis at presentation, making it a unique case in the literature. This tumor's pattern of spread can be considered analogous to that of a stage II ovarian cancer—defined as tumor extension into the fallopian tubes, uterus, or pelvic tissues [9].

A high-quality systematic review of adjuvant therapy for stage I/IIa ovarian cancer showed that platinum-based adjuvant chemotherapy improved survival and reduced risk of recurrence compared to those who were only treated surgically [6]. The current GCIG consensus is that a taxane plus a platinum-based agent should be the comparator treatment in control arms of ovarian cancer clinical trials [10]. For this reason, the combination of paclitaxel and carboplatin was selected in this case. Although our patient’s current disease-free status following this regimen is certainly encouraging, determining whether or not the addition of adjuvant chemotherapy provides a measurable benefit in cases of high-grade, nonmetastatic testicular serous papillary carcinoma will only be possible once a larger population of patients with this disease are treated accordingly.

## Conclusions

Due to the rarity of ovarian-type testicular tumors and lack of evidence regarding outcomes, developing an evidence-based treatment plan is challenging for this disease. Serous papillary carcinoma of the paratestis is extremely rare, but histologically similar to its more common ovarian counterpart. The case we present here demonstrates that it may be prudent to take advantage of the existing literature for ovarian cancer, and to consider adjuvant chemotherapy for high-grade disease.
